# The Hypoglycemic and Antioxidant Activity of Cress Seed and Cinnamon on Streptozotocin Induced Diabetes in Male Rats

**DOI:** 10.1155/2016/5614564

**Published:** 2016-07-21

**Authors:** Safaa Qusti, Haddad A. El Rabey, Sarah A. Balashram

**Affiliations:** ^1^Biochemistry Department, Faculty of Science, King Abdulaziz University, Jeddah 21789, Saudi Arabia; ^2^Bioinformatics Department, Genetic Engineering and Biotechnology Institute, Sadat City University, Sadat City, Monufia 32897, Egypt

## Abstract

The present study aimed to estimate the stimulation of pancreas of rats with streptozotocin induced diabetes using 20% (w/w) garden cress seed (*Lepidium sativum*) and cinnamon methanol extracts. The positive control diabetic group showed a significant increase in fasting blood sugar, lipid peroxide, interleukin-6, carboxymethyl lysine, serum uric acid, urea, creatinine, immunoglobulins, and urine albumin and a significant decrease in antioxidant enzymes, sodium ions, potassium ions, and urine creatinine. Severe histopathological changes in the kidney and pancreas tissues in hyperglycemic rats were also shown in the positive control diabetic group. Meanwhile, the groups that were treated with 20% garden cress seed and cinnamon methanol extracts showed a significant decrease in fasting blood sugar and all elevated abovementioned biochemical parameters and an increase in the lowered ones restoring them nearly to the normal levels of G1. Kidney and pancreas tissues were also ameliorated and restored nearly to the normal status. Both garden cress seed and cinnamon methanol extracts succeeded in controlling hyperglycemia in rats with streptozotocin induced diabetes and ameliorated the biochemical and histopathological changes because of their antioxidant activity acquired by their possession of phenolic phytochemicals.

## 1. Introduction

Diabetes mellitus (DM) is a metabolic disorder characterized by chronic hyperglycemia linked with total or partial deficiencies in insulin secretion or function. It is one of the most frequent chronic diseases affecting millions of people globally leading to morbidity and mortality worldwide particularly in developing countries of Africa, Asia, and South America [1–3]. Diabetes mellitus is considered an extended chronic metabolic disease that causes several other complications, such as cardiovascular diseases; fixed cost used for its treatment placed a huge burden on the economy and health systems worldwide [4, 5].

Traditional medicines and plant-based systems continue to play an essential role in healthcare [6]. Cinnamon and garden cress seeds are members of a list containing 150 plants used in treatment of diabetes mellitus [7]. Cinnamon is a spice obtained from the inner bark of several trees from the genus* Cinnamomum* (family: Lauraceae) [8]. The active compounds of cinnamon have been reported, such as water-soluble polyphenol type-A polymers, cinnamaldehyde, and cinnamic acid [1]. It is used for treating abdominal and chest pains, chronic diarrhea, hypertension, kidney disorders, and rheumatism [9]. Cinnamon extracts were shown to have antidiabetic effects as a number of cell studies demonstrated an insulin-like action. Additionally, cinnamaldehyde promoted glucose uptake into skeletal muscle through glucose transporter 4 translocation [10].

*Lepidium sativum* L. (garden cress) contains mucilage in its dry seed coat that has been isolated using dissimilar solvents and utilized by researchers as an excipient in a variety of pharmaceutical formulations for preferred functionality [11]. Garden cress seed mucilage is extensively used in many traditional medicinal arrangements such as cough syrups. It also has antihyperglycemic properties which help to control glucose level in diabetics [12, 13]. The seeds of* L*.* sativum* are aperient, diuretic, tonic, demulcent, carminative, galactagogue, and emmenagogue, are used to induce an abortion, and also possess antibacterial and antifungal properties [14].

This study aimed to estimate the stimulation of the pancreas by the antidiabetic effect of 20% (w/w) garden cress seed (*Lepidium sativum*) and cinnamon methanol extracts in male rats with STZ induced diabetes.

## 2. Materials and Methods

The experimental work of the present study was conducted at king Fahd Medical Research Center and Faculty of Science, King Abdulaziz University, Jeddah, Saudi Arabia.

### 2.1. Materials

Cinnamon and garden cress seed were purchased from a local herbal medicine shop in Jeddah, Saudi Arabia.

### 2.2. Conventional Animal Basal Diet

The conventional animal basal diet was obtained from a grain mill in Jeddah. Every 100 g consists of the following: 12% protein (17.14 g of 70% casein), 4 g corn oil (4% fat), 0.3 g methionine (0.3%), 0.2 g choline chloride (00.2%), 4 g minerals (4% minerals), 1 g vitamin mixture (1% vitamin), 4 g cellulose (4% fiber), and 69.36 g corn starch (69.36%). The basal diet was stored in a dry place away from direct sunlight.

### 2.3. Animals

Forty adult male albino rats weighing 180 to 200 g were used in this study. All animal experiments were carried out under protocols approved by the Institutional Animal House of the University of King Abdulaziz at Jeddah, Saudi Arabia. The animals were housed in cages and received normal rat chow and tap water* ad libitum* in a constant environment (room temperature 28 ± 2°C, room humidity 60 ± 5%) with a 12 h light and 12 h dark cycle. The animals were kept under observation for two weeks prior to the start of the experiments.

### 2.4. Study Design

Ten rats were used as control group (the first group, G1) and received a single tail vein injection of 0.1 mol/L citrate buffer only. The other 30 rats were intravenously injected with freshly prepared streptozotocin (65 mg/kg body weight) in a 0.1 mol/L citrate buffer (pH 4.5), after fasting for 12 hours to induce diabetes [15]. After five days of injection, rats with blood glucose higher than 200 mg/dL were considered diabetic in the fasting state. Rats with blood glucose lower than 200 mg/dL were excluded from the study. Glucose measurement was done by using* OneTouch Select* Analyzer (LifeScan, Inc., UK). The study was started one week after STZ injection. The 30 diabetic rats were randomly divided into 3 groups. The second group (G2) was the diabetic control positive group fed normal basal diet. The third group (G3) was diabetic group treated with 20% (w/w) garden cress seeds methanol extract, orally using stomach tube for 28 days. The fourth was diabetic group treated with 20% (w/w) cinnamon methanol extract, orally using stomach tube for 28 days.

### 2.5. Phytochemical Analysis

The total flavonoid contents of cinnamon and garden cress seed were determined by a colorimetric method as follows: each sample (0.5 mL) was mixed with 2 mL of distilled water and subsequently with 0.15 mL of a NaNO_2_ solution (15%). After 6 minutes, 0.15 mL of aluminum chloride (AlCl_3_) solution (10%) was added and allowed to stand for 6 minutes, and then 2 mL of NaOH solution (4%) was added to the mixture. Immediately, water was added to bring the final volume to 5 mL and the mixture was thoroughly mixed and allowed to stand for another 15 minutes. Absorbance of the mixture was then determined at 510 nm versus prepared water blank.

Total phenol estimation was carried out using Folin-Ciocalteu reagent according to the method of Malick and Singh [16]. Phenols react with phosphomolybdic acid in Folin-Ciocalteu reagent in alkaline medium and produce blue-colored complex. The absorbance of mixture was measured using spectrophotometer at 650 nm against a reagent blank. A standard curve was prepared using different concentrations of catechol and then total phenols were expressed as g of phenols/100 g material. Total carotenoids were extracted with acetone-hexane mixture and determined with a spectrophotometer at wave length 440 nm as described by Dubois et al. [17].

### 2.6. Preparation of Methanol Extract

Cinnamon and garden cress seed were milled by mixer, and then methanol extract was prepared according to the method of Adebayo et al. [18] as follows: 200 g of each of cinnamon and garden cress seed was soaked in 1 liter of 90% methyl alcohol under shaking for 5 days and kept in a refrigerator. The methanol was evaporated using a rotatory evaporator apparatus attached with a vacuum pump. Twenty grams of either extract (semisolid) was suspended in 100 mL distilled water with 2 mL of tween 80 (suspending agent) to prepare a 20% alcoholic solution.

### 2.7. Samples Collection

At the end of the experiment, rats were fasted 14–16 hours after their last feeding and blood samples were collected from the heart of each rat under anesthesia with diethyl ether. Blood sample of rats was centrifuged at ×2,000 g for 10 minutes at 4°C, and serum was removed and stored at −80°C until analysis.

### 2.8. Urine Sample

Before induction of diabetes and the day before the end of the experiment, urine samples were collected by placing the rats in individual metabolic cages for 24 h.

### 2.9. Dissection

Animals were sacrificed using ether anesthesia by cervical dislocation, and then the abdomen was dissected and heart, liver, right kidney, left kidney, left testis, and right testis were dissected and weighed. In addition, one kidney and a piece of the pancreas were saved in saline buffer (0.9% NaCl) for histopathological investigations and the other kidney was kept in ice for homogenate preparation.

### 2.10. Kidney Homogenate Preparation

Kidney tissue was cut into small pieces and washed by phosphate-buffered saline and then ground in a homogenization buffer, and then the homogenate was prepared as described in Al-Malki and El Rabey [19]. The homogenate was used for the determination of reduced glutathione (GSH), level of lipid peroxidation (MDA), concentration of* Nε*-carboxymethyl lysine (CML), activity of superoxide dismutase (SOD), and levels of IL-6. The other kidney from each group was used for histopathological examination.

### 2.11. Determination of Glucose

Glucose was assayed using a kit from Human (Germany) according to the method of Barham and Trinder [20].

### 2.12. Determination of Carboxymethyl Lysine (CML)

Carboxymethyl lysine (CML) was estimated in the serum according to the method of Monnier and Cerami [21] using ELISA kits from MyBIOSOURCE (Canada). This kit employs Double Antibody Sandwich Technique. The principle of Double Antibody Sandwich is based on characteristics of the tested antigen with more than two valances which can identify coated antibody and detection antibody at the same time.

### 2.13. Determination of Interleukin-6 (IL-6)

Interleukin-6 (IL-6) was estimated in the serum and the kidney tissue homogenate by Sayed [15] using immunoassay kit from R&D Systems Inc. (USA).

### 2.14. Antioxidants Enzymes Activity

Superoxide dismutase (SOD) activity was estimated in the serum and in the kidney tissue homogenate according to the method described by Nishikimi et al. [22] using colorimetric kit from Biodiagnostic Chemical Company (Egypt). Catalase (CAT) activity was estimated in the serum and in the kidney tissue homogenate according to the method described by Aebi [23] using colorimetric kit from Biodiagnostic Chemical Company (Egypt). Glutathione-S-transferase (GST) was estimated in the serum and in the kidney tissue homogenate according to the method described by Habig et al. [24] using a special kit from Biodiagnostic Chemical Company (Egypt).

### 2.15. Determination of Lipid Peroxidation (MDA)

Lipid peroxidation was assayed in the serum and in the kidney tissue homogenate using a kit from Biodiagnostic Chemical Company (Egypt) according to the method of Ohkawa et al. [25] according to the instructions of the supplier.

### 2.16. Kidney Functions

Uric acid was estimated according to Barham and Trinder [20] using enzymatic colorimetric kit, PAP-method from Human (Germany). Creatinine was assayed in the serum and in the urine by Bartels et al. [26] using a photometric colorimetric kit, Jaffe reaction from Human (Germany). Urea was estimated in the serum according to the method described by Berthelot [27] and Fawcett and Scott [28] using enzymatic colorimetric kit from Human (Germany).

### 2.17. Determination of Electrolytes

Sodium (Na^+^) was assayed using a kit from Human (Germany) according to the colorimetric method of Trinder [29]. Potassium (K^+^) was estimated according to the method of Terri and Sesin [30] using Human (Germany).

### 2.18. Urine Analysis

Creatinine and albumin were estimated in urine. Urine albumin was estimated using a Nephrat II Albumin Kit from Exocell Inc., Philadelphia, PA, USA, according to the method of Sayed [15].

### 2.19. Determination of Immunoglobulins

Immunoglobulins (IgA, IgM, and IgG) were estimated in the serum according to Berne [31] using commercially available kits from Genway Biotech (USA) according the instruction of the suppliers.

### 2.20. Physiological Parameters

The following physiological parameters were estimated according to the method of Davies and Morris [32] as follows:

- Food intake and water consumption were calculated every week.
- Total body weight: rats were weighed every week.
- Food intake (FI) body weight gain (BWG) and food efficiency ratio (FER) were calculated.
- Heart, liver, right kidney, left kidney, left testis, and right testis were weighed after dissection and the relative organ weight was calculated by dividing the organ weight on the total body weight of each rat and then multiplied by 100.

### 2.21. Histopathological Examination

Five *μ*m thick sections of kidney and pancreatic tissues were prepared and stained with hematoxylin and eosin (H&E) dye for microscopic investigation according to Drury et al. [33]. The stained sections were examined and photographed under an Olympus light microscope with a digital camera.

### 2.22. Statistical Analysis

Values were analyzed using SPSS program to calculate the *t*-test and the mean ± SD and then analyzed using one-way analysis of variance (ANOVA, *P* < 0.05) using a protected least significant difference (LSD) test of SAS package.

## 3. Results

### 3.1. Phytochemical Analysis of Cress Seeds and Cinnamon

Table 1 shows the phytochemical analysis of* L*.* sativum* and cinnamon.* L*.* sativum* seeds that contain 58.8 mg/100 g DW total phenols, 42.35 mg/100 g DW flavonoids, and 1.43 mg/100 g DW carotenoids, whereas cinnamon contains 52.7 mg/100 g DW total phenols, 1235 mg/100 g DW flavonoids, and 567.3 mg/100 g DW carotenoids.

### 3.2. Fasting Blood Sugar, CML, and IL-6

Table 2 shows the effect of administration of* L*.* sativum* and cinnamon methanol extracts for 4 weeks on serum fasting blood sugar, CML, and IL-6 in diabetic rats. Induction of diabetes in the positive control group (G2) significantly (*P* < 0.001) increased the mean values of serum fasting blood sugar in the positive control compared with that of the negative control. Treating the diabetic rats with* L*.* sativum* and cinnamon methanol extracts in G3 and G4, respectively, significantly (*P* < 0.001) decreased the mean values of serum fasting blood sugar compared with the positive control. In addition, treating the diabetic rats in G4 with cinnamon methanol extract was much more efficient than* L*.* sativum* methanol extract in G3.

Table 2 also shows the effect of administration of* L*.* sativum* and cinnamon methanol extracts for 4 weeks on carboxymethyl lysine (CML) in diabetic rats. The mean values of carboxymethyl lysine in the positive control were significantly (*P* < 0.001) higher than that of the negative control. Treating the diabetic rats with* L*.* sativum* and cinnamon methanol extracts in G3 and G4, respectively, significantly (*P* < 0.001) decreased the mean values of CML compared to that of the positive control. Treating diabetic rats with cinnamon methanol extract in G4 was more efficient than treating with* L*.* sativum* in G3.

Table 2 also shows the effect of administration of* L*.* sativum* and cinnamon methanol extracts for 4 weeks on serum interleukin-6 (SIL-6) and kidney tissue homogenate (TIL-6) of diabetic rats. The mean values of serum IL-6 and kidney tissue homogenate in the positive control were higher than that of the negative control. Treating the diabetic rats in G3 and G4 with* L*.* sativum* and cinnamon methanol extract significantly (*P* < 0.001) decreased the mean values of SIL-6 and TIL-6 more than that of the positive control. Cinnamon methanol extract (in G4) was much more efficient than* L*.* sativum* methanol (in G3) extract in lowering SIL-6 and TIL-6.

### 3.3. Antioxidants Enzymes

Table 3 shows the effect of administration of* L*.* sativum* and cinnamon methanol extracts for 4 weeks on antioxidants enzymes in the serum and kidney tissue homogenate of diabetic rats. The mean values of catalase (CAT), superoxide dismutase (SOD), and glutathione S-transferase (GST) in serum and kidney tissue homogenate of the positive control were very high and significantly (*P* < 0.001) decreased compared with that of the negative control. Treating the diabetic rats in G3 and G4 with* L*.* sativum* and cinnamon methanol extract significantly (*P* < 0.001) increased the mean values of CAT, SOD, and GST compared with that of the positive control. Generally, treating the diabetic rats with cinnamon methanol extract in G4 increased all the studied antioxidant enzymes in serum more than* L*.* sativum* methanol extract in G3.

### 3.4. Lipid Peroxide

Table 4 shows the effect of administration of* L*.* sativum* and cinnamon methanol extracts for 4 weeks on lipid peroxide in diabetic rats. The mean values of MDA in the serum and kidney tissue homogenate of the positive control were significantly (*P* < 0.001) higher than that of the negative control. Treating the diabetic rats with* L*.* sativum* and cinnamon methanol extracts for 4 weeks in G3 and G4, respectively, very highly significantly (*P* < 0.001) lowered the mean values of lipid peroxide compared to that of the positive control. It is worth mentioning that treating diabetic rats with cinnamon methanol extract in G4 was more efficient than* L*.* sativum* extract in G3.

### 3.5. Renal Function

Table 5 shows the effect of administration of* L*.* sativum* and cinnamon methanol extracts for 4 weeks on serum urea, creatinine, uric acid, and sodium and potassium ions in diabetic rats. Induction of diabetes significantly (*P* < 0.001) increased the mean values of serum urea, creatinine, and uric acid in the positive control compared with that of the negative control due to the diabetic nephropathy. Treating the diabetic rats in G3 and G4 with* L*.* sativum* and cinnamon methanol extract, respectively, significantly (*P* < 0.001) decreased the mean values of all renal function parameters compared with that of the positive control.

Table 5 also shows the effect of administration of* L*.* sativum* and cinnamon methanol extracts for 4 weeks on sodium and potassium ions in the serum of diabetic rats. The mean values of sodium and potassium ions in serum of the positive control were significantly (*P* < 0.001) lower than that of the negative control. Treating the diabetic rats with* L*.* sativum* and cinnamon methanol extracts in G3 and G4, respectively, significantly (*P* < 0.001) increased the mean values of sodium and potassium ions in the serum compared with that of the positive control.

### 3.6. Urine Analysis

Table 6 shows the effect of administration of* L*.* sativum* and cinnamon methanol extracts for 4 weeks on urine albumin and creatinine in diabetic rats. The mean values of urine albumin in the positive control were significantly (*P* < 0.001) higher than that of the negative control. Treating the diabetic rats in G3 and G4 significantly (*P* < 0.001) decreased the mean values of urine albumin compared to that of the positive control.

The mean values of urine creatinine in the positive control were significantly (*P* < 0.001) lower than that of the negative control. Treating the diabetic rats in G3 and G4* L*.* sativum* and cinnamon methanol extracts for 4 weeks significantly (*P* < 0.001) increased the mean values of urine creatinine compared to that of the positive control.

### 3.7. Immunoglobulins (IgG, IgA, and IgM)

Table 7 shows the effect of administration of* L*.* sativum* and cinnamon methanol extracts for 4 weeks on serum immunoglobulins (IgG, IgA, and IgM) in diabetic rats. Induction of diabetes in G2 significantly (*P* < 0.001) increased the mean values of the serum IgG, IgA, and IgM in the positive control compared with that of the negative control. Treating the diabetic rats with* L*.* sativum* and cinnamon methanol extracts in G3 and G4, respectively, significantly (*P* < 0.001) decreased the mean values of IgG, IgA, and IgM compared with that of the positive control. Moreover, treating diabetic rats with cinnamon methanol extract in G4 was more efficient in ameliorating immunoglobulins more than treating them with* L*.* sativum* in G3.

### 3.8. Total Body Weight

Table 8 shows the effect of administration of* L*.* sativum* and cinnamon methanol extracts for 4 weeks on the total body weight in diabetic rats under study. The mean values of total body weight of the positive control in the 1st, 2nd, 3rd and 4th weeks were significantly (*P* < 0.01) lower than that of the negative control. Treating the diabetic rats in G3 and G4 significantly (*P* < 0.01) increased the mean values of total body weight compared with that of the positive control. Moreover, treating diabetic rats with cinnamon methanol extract in G4 was more efficient in ameliorating immunoglobulins more than treating them with* L*.* sativum* in G3.

### 3.9. Food Intake

Table 9 shows the effect of administration of* L*.* sativum* and cinnamon methanol extracts for 4 weeks on food intake in diabetic rats. In the 1st, 2nd, 3rd and 4th weeks, the mean values of food intake in all groups were approximately equal. The differences were nonsignificant.

### 3.10. Water Consumption

Table 10 shows the effect of administration of* L*.* sativum* and cinnamon methanol extracts for 4 weeks on water consumption in diabetic rats. Water consumption was nonsignificantly affected as a result of diabetes for all groups in all weeks except for the 2nd and 3rd week of the positive control (G2) and G3 and G4 of the 1st week.

### 3.11. Organs Weight

Table 11 shows the effect of administration of* L*.* sativum* and cinnamon methanol extracts for 4 weeks on organ weight in diabetic rats. The mean values of heart weight in the positive control were nonsignificantly higher than that of the negative control in all weeks, whereas treating the diabetic rats in G3 and G4 with cress seed and cinnamon methanol extract significantly increased all organ weights in all weeks.

### 3.12. Physiological Evaluation

Table 12 shows the effect of administration of* L*.* sativum* and cinnamon methanol extracts for 4 weeks on food intake, body weight gain (BWG), and food efficiency ratio (FER) in diabetic rats. The mean values of body weight gain (BWG)/day, BWG g/4 weeks, and BWG% in the positive control were lower than that of the negative control, whereas treating the diabetic rats with cress seed and cinnamon methanol extract significantly increased these BWG parameters. On the other hand, FER and FER% in the positive control group were significantly lower than that of the negative control. In G3 and G4, the mean values of FER and FER% were higher than those of the positive control.

### 3.13. Histopathology of Kidney

**Figure 1:**
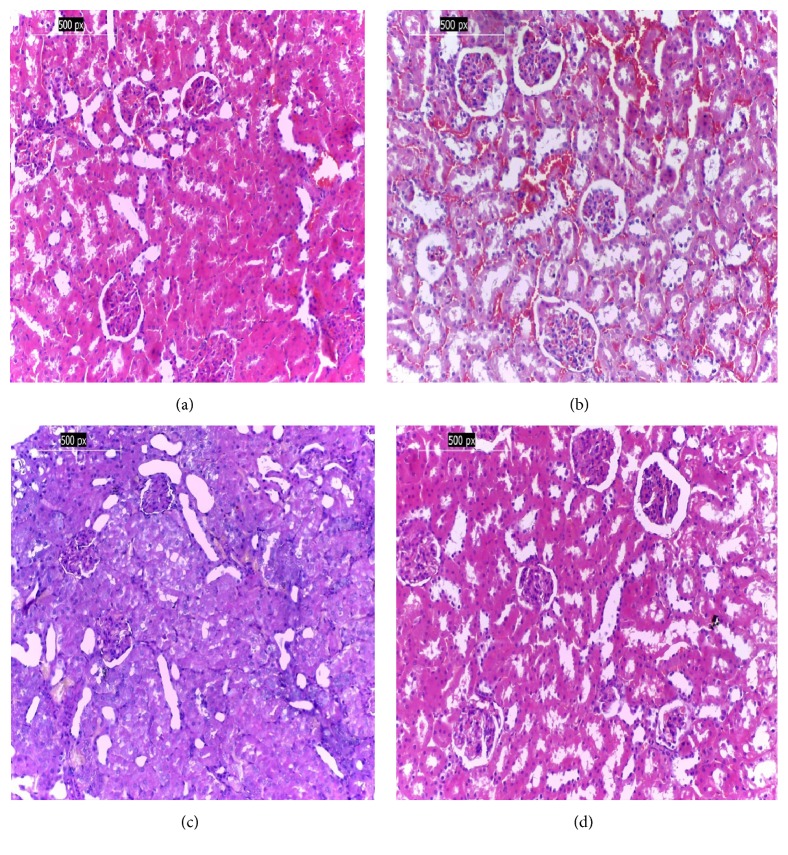
(a) Photomicrography of a kidney of negative control group (G1) reveals normal histological structure; (b) photomicrography of a kidney of the positive control group with pathological changes; (c) photomicrography of a kidney of G3 treated with* L*.* sativum* shows nearly normal tissues; (d) photomicrography of a kidney of G4 group treated with cinnamon shows nearly normal renal cortical tissue (H&E ×200).

Kidney of the negative control group (G1) exhibited the general structure of normal kidney with normal histological structure of renal parenchyma, normal renal tissue, blood vessels, and normal interstitial tissue with no histopathological changes (Figure 1(a)). The positive control (G2) group shows collapsed glomerular tuft with marked tubular atrophy interstitial inflammation and interstitial hemorrhage (Figure 1(b)). The kidney of diabetic rats in the third group (G3) that were treated with* L*.* sativum* shows normal glomeruli regenerated tubule with persistent interstitial hemorrhage (Figure 1(c)). Similarly, the kidney tissue of a rat from group G4 treated with cinnamon (Figure 1(d)) shows near normal renal cortical tissue.

### 3.14. Histopathology of Pancreas

**Figure 2:**
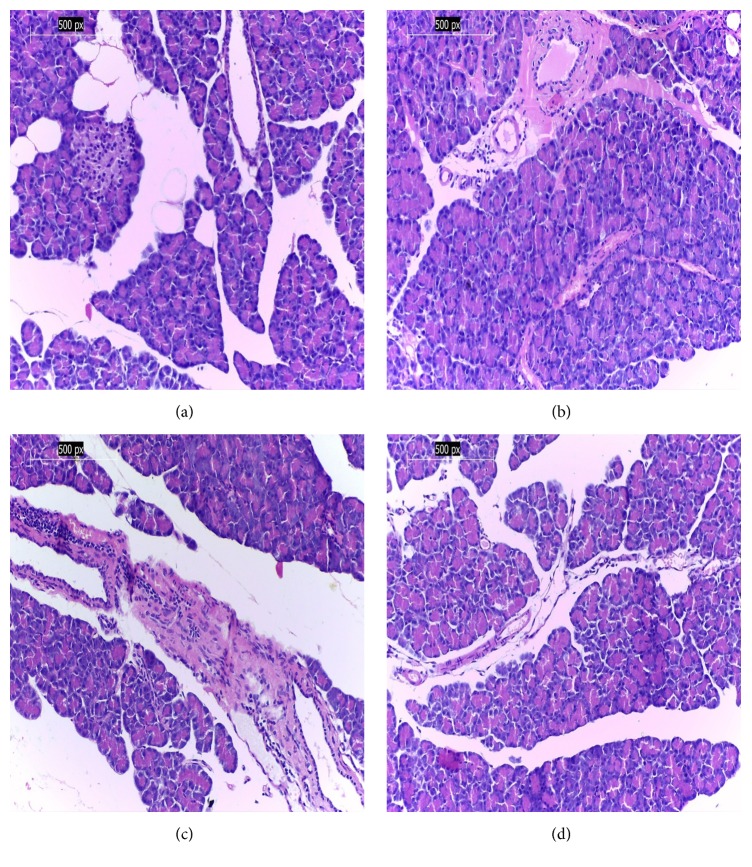
(a) Photomicrography of pancreas of rat from the negative control group showing normal pancreatic islets and glands; (b) photomicrography of pancreas of rat from the positive control group shows sever pathological changes; (c) photomicrography of pancreas of rat from G3 treated with* L*.* sativum* with very mild inflammation and edema; (d) photomicrography of pancreas of rat from group G4 treated with cinnamon shows normal pancreatic tissue (H&E ×200).

The histology of pancreas is shown in Figure 2. Pancreas tissues of rat from negative control group showing normal pancreatic acini Langerhans cells and interductal glands are shown in Figure 2(a). Pancreas tissues of the positive control diabetic group (G2) showed mild degeneration of pancreatic acini cells with periductal inflammation and edema with mild congestion (Figure 2(b)).  Figure 2(c) shows pancreas tissues of rat from group G3 that were treated with* L*.* sativum* for 4 weeks with very mild inflammation and edema.  Figure 2(d) shows pancreas of rat from group G4 treated with cinnamon for 4 weeks with normal pancreatic tissue.

## 4. Discussion

This study was conducted in order to find out the antidiabetic activity of* L*.* sativum* and cinnamon methanol extract in rats with STZ induced diabetes. Induction of diabetes increased the fasting blood sugar in the diabetic male rats of the positive control group. The phenolic, flavonoid, and carotenoid content of both garden cress and cinnamon encouraged us to test their antioxidant potential in controlling STZ induced diabetes and diabetic nephropathy as one of diabetic complications. The flavonoid group of compounds in* L*.* sativum* has anti-inflammatory activity [34]. Phenolic phytochemicals have antioxidative, antidiabetic, anticarcinogenic, antimicrobial, antiallergic, antimutagenic, and anti-inflammatory activities [35].

The current results showed a decrease in the levels of FBS in G3 and G4 after treatment with* L*.* sativum* and cinnamon methanol extract, respectively, for 4 weeks compared with the positive control group. This result is consistent with that of Abdelwahab et al. [8] using the aqueous* L*.* sativum* extract that significantly reduced the blood glucose levels after a single or repeated administration. The strong hypoglycemic action of* L*.* sativum* extract is due to the presence of benzyl isothiocyanate [1, 11]. Similarly, cinnamon acquired its antidiabetic activity because it contains several phenolic compounds as catechin, epicatechin, procyanidin B2, and phenol polymers that showed significant inhibitory effects on the formation of advanced glycation end products [36]. It also possesses insulin mimetic properties because its biologically active substances enhance glucose uptake by activating insulin receptor kinase activity, autophosphorylation of the insulin receptor, and glycogen synthase activity [37].

In the current study, interleukin-6 levels were increased in the serum and kidney tissue homogenate as a result of diabetes in G2 as a result of STZ induced diabetes. Treating the diabetic rats with* L*.* sativum* and cinnamon methanol extracts showed a significant decrease in levels of interleukin-6 in G3 and G4 compared with the positive control. Several inflammatory cytokines including TNF-a, IL-1b, and IL-6 have been identified as being involved in the development of insulin resistance [19, 38]. Their interference with insulin signaling leads to hyperglycemia and proinflammatory changes. IL-6 influences insulin sensitivity by directly impairing insulin signaling in primary mouse hepatocytes and 3T3-L1 adipocytes with decreased activity. In hamsters with fructose feeding induced insulin-resistant diabetes, the serum levels of TNF-a and IL-6 were found to be significantly higher compared with those of the chow-fed hamsters [39].

In the current study, the levels of carboxymethyl lysine which is an advanced glycation end product (AGE) were increased in the serum as a result of diabetes in G2. Treating the diabetic rats with* L*.* sativum* and cinnamon methanol extracts showed a significant decrease in CML levels in G3 and G4 compared with the positive control. Adisakwattana et al. [40] indicated that cinnamic acid and its derivatives significantly inhibit the formation of advanced glycation end products (AGEs) by approximately 11.96–63.36% in a concentration-dependent manner.

Our result showed a significant decrease in the levels of catalase, SOD, and GST in serum and kidney tissue homogenate of the positive control group (G2) compared with that of the negative control group (G1) as a result of induction of diabetes. This result is consistent with Eidi et al. [41] and Al-Malki and El Rabey [19]. In addition, Baynes and Thorpe [42] reported that hyperglycemia increases the generation of free radicals by glucose autooxidation and the increment in free radicals thereby depleting the activity of antioxidant defense system and thus promoting de novo free radical generation that may lead to liver cell damage. However, in G3 and G4 the levels of catalase, SOD, and GST in serum and kidney tissue homogenate was increased compared with the positive control group as a result of treatment of diabetic rats with* L*.* sativum* or cinnamon methanol extract for 4 weeks. Both* L*.* sativum* and cinnamon contain polyphenols, which are among the natural dietary antioxidants found in cinnamon and have been shown to reduce oxidative stress via the inhibition of 5-lipoxygenase [43].

The current study showed that lipid peroxide was increased in serum and kidney tissue homogenate as a result of diabetes induction in the positive control group (G2). This result is in agreement with that of Al-Malki and El Rabey [19]. Meanwhile, after treatment with* L*.* sativum* or cinnamon in G3 and G4, respectively, a significant decrease in the levels of MDA was encountered compared with the positive control. Cinnamon contains high level of phenolic groups that cause scavenging of free radicals which is one of the major antioxidation mechanisms to inhibit the chain reaction of lipid peroxidation [44]. In addition, our results concerning the elevation of MDA and reduction in antioxidants enzyme activity due to induction of diabetes and amelioration after* L*.* sativum* and cinnamon treatments are consistent with other studies [36].

The renal function showed that an increase in serum urea, creatinine, uric acid, and urine albumin was disrupted by diabetes induction in the positive control group (G2). This result is consistent with the fact that STZ induced diabetes leads to diabetic nephropathy and consistent with the studies of Sayed [15] and Al-Malki and El Rabey [19]. Treating the diabetic rats of G3 and G4 with* L*.* sativum* and cinnamon methanol extract, respectively, showed a significant decrease in serum urea, creatinine, uric acid, and urine albumin and increase in urine creatinine. This result agrees with that of Mogensen and Christensen [45] and Kumar et al. [46]. This amelioration in renal function is due to the presence of flavonoids and steroidal compounds [46]. The current results also showed significant decrease in serum sodium and potassium ions level as a result of diabetes, whereas the methanol extracts of cinnamon and cress seeds restored them to the normal levels. Cinnamon has the most potent inhibitory effect on the intestinal ATPase as compared to extracts of other spices [47]. They also inhibited the* in vitro* Na^+^-K^+^-ATPase activity in a crude kidney homogenate and the activity of an isolated dog kidney Na^(+)^-K^(+)^-ATPase [48, 49].

The immunoglobulins (IgG, IgA, and IgM) results showed a significant increase in immunoglobulins (IgG, IgA, and IgM) compared with the negative control group. Treating the diabetic rats in G3 and G4 with cress seed and cinnamon methanol extract, respectively, restored these immunoglobulins to the normal levels. This result is consistent with that of Muthenna et al. [50] who stated that cinnamon is able to prevent cross-linking of IgG on red blood cell surface (RBC-IgG). During diabetic conditions, there is a considerable increase in RBC-IgG cross-linking that provides an index of AGE mediated protein cross-linking. Cinnamon obtained from twigs of* Cinnamomum osmophloeum* contains oils reported by Tung et al. [51] to have powerful anti-inflammatory properties. The essential oils and major constituents are primarily represented by trans-cinnamaldehyde, caryophyllene oxide, L-borneol, L-bornyl acetate, eugenol, beta-caryophyllene, E-nerolidol, and cinnamyl acetate. These oils were able to reduce chronic inflammation in granulomatous responses [52].

The total body weight (g) in G2 showed a significant decrease as a result of induction of diabetes, whereas it increased with treating with cinnamon and cress seed methanol extract. This result is consistent with that of Beejmohun et al. [53]. In our study, weight of heart, testes, left kidney, and liver in all groups showed a significant increase as a result of diabetes, whereas the right kidney showed the no significant change. Restoring the normal organ's weight as a result of treating diabetic rats with cinnamon and cress seed methanol extract is consistent with Elgawish and Abdelrazek [54]. For food intake, our result showed no significant change in all groups and the values were very close. Similar results by Torki et al. [55] stated that food intake and body weight were not affected by dietary zinc and cinnamon essential oil.

Water consumption showed no significant changes in all groups, except the first week that showed significant increase in G3 and G4 rats compared with the control group. For the physiological evaluation our result showed significant increase in BWG g/day, BWG g/4 week, BWG%, FER g/day, and FER% compared with the control group. The result of Al-Yahya et al. [56] agrees with our result.

The histological studies showed altered pathological changes in the tissues of kidney and pancreas as a result of diabetes in the positive control group [1, 19], whereas treating the diabetic rats with cress seed and cinnamon methanol extract restored the altered tissues nearly to the normal conditions. Ullah et al. [57] stated that cinnamon significantly attenuated aminoglycosides-kidney toxicity by improving the urea, creatinine, uric acid, urinary protein levels, and histopathological alterations of the kidneys. In addition, our result is in agreement with that of Al-Malki and El Rabey [19].

It could be concluded that both cress seed (*Lepidium sativum*) and cinnamon extracts methanol extracts succeeded in controlling hyperglycemia in rats with streptozotocin induced diabetes. They also ameliorated all biochemical tests and kidney and pancreas functions and tissues and restored them to the normal state because of their antioxidant activity acquired by their possession of phenolic phytochemicals.

## Figures and Tables

**Table 1:** The phytochemical analysis of *L. sativum* and cinnamon as revealed by spectrophotometric analysis.

Material	Total phenols	Flavonoids	Carotenoids
*L. sativum*	58.8 mg/100 g	42.35 mg/100 g	1.43 mg/100 g
Cinnamon	52.7 mg/100 g	1235 mg/100 g	567.3 mg/100 g

**Table 2:** Effect of administration of *L. sativum* and cinnamon methanol extracts for 4 weeks on blood sugar, CML, and IL-6 in diabetic rats.

	Statistics	G1 Negative control	G2 Positive control	G3 *L. sativum* extract	G4 Cinnamon extract
FBS mg/dL	Mean ± SE	92.666 ± 1.145^d^	283.333 ± 2.472^a^	206.333 ± 2.444^b^	147.000 ± 1.211^c^
	LSD 0.05 = 6.256				
	*t*-test	—	−63.63^*∗∗∗*^	16.67^*∗∗∗*^	53.15^*∗∗∗*^

Carboxymethyl lysine (CML)%	Mean ± SE	188.16 ± 2.38^d^	276.00 ± 2.58^a^	243.66 ± 1.72^b^	227.16 ± 1.30^c^
	LSD 0.05 = 6.415				
	*t*-test	—	−24.84^*∗∗∗*^	13.53^*∗∗∗*^	16.07^*∗∗∗*^

Serum interleukin-6 (SIL-6) pg/mL	Mean ± SE	5.600 ± 0.260^d^	24.483 ± 0.892^a^	15.883 ± 0.612^b^	8.733 ± 0.230^c^
	LSD 0.05 = 1.864				
	*t*-test	—	−17.24^*∗∗∗*^	6.55^*∗∗∗*^	21.77^*∗∗∗*^

TIL6 pg/mL	Mean ± SE	48.800 ± 2.010^d^	90.433 ± 1.551^a^	74.216 ± 0.772^b^	63.966 ± 1.275^c^
	LSD 0.05 = 4.276				
	*t*-test	—	−14.14^*∗∗∗*^	16.14^*∗∗∗*^	12.93^*∗∗∗*^

Data are represented as mean ± SE. For *t*-test values, *∗∗∗* is significant at *P* < 0.001. For ANOVA analysis, within each row, means with different superscript (a, b, c, or d) are significantly different at *P* < 0.05, whereas means superscripts with the same letters mean that there is no significant difference at *P* < 0.05. LSD: least significant difference.

**Table 3:** Effect of administration of *L. sativum* and cinnamon methanol extracts for 4 weeks on antioxidants enzymes in the serum of diabetic rats.

Parameters serum	Statistics	G1 Negative control	G2 Positive control	G3 *L. sativum* extract	G4 Cinnamon extract
Catalase (CAT) U/I	Mean ± SE	2.405 ± 0.193^a^	0.151 ± 0.011^d^	0.950 ± 0.036^c^	1.808 ± 0.016^b^
	LSD 0.05 = 0.295				
	*t*-test	—	11.49^*∗∗∗*^	−24.48^*∗∗∗*^	−73.76^*∗∗∗*^

Superoxide dismutase (SOD) U/mL	Mean ± SE	638.683 ± 1.561^a^	120.833 ± 2.411^d^	239.683 ± 1.597^c^	487.016 ± 1.469^b^
	LSD 0.05 = 5.922				
	*t*-test	—	178.65^*∗∗∗*^	−37.04^*∗∗∗*^	−168.71^*∗∗∗*^

Glutathione reductase (GSST) U/mL	Mean ± SE	813.200 ± 2.320^a^	120.933 ± 2.381^d^	239.683 ± 1.597^c^	714.250 ± 2.478^b^
	LSD 0.05 = 7.090				
	*t*-test	—	228.70^*∗∗∗*^	−37.37^*∗∗∗*^	−169.43^*∗∗∗*^

Catalase (CAT) U/g kidney tissue	Mean ± SE	5.028 ± 0.085^a^	0.385 ± 0.023^d^	1.495 ± 0.065^c^	2.720 ± 0.056^b^
	LSD 0.05 = 0.205				
	*t*-test	—	51.41^*∗∗∗*^	−14.99^*∗∗∗*^	−37.78^*∗∗∗*^

Superoxide dismutase (SOD) U/g. kidney tissue	Mean ± SE	917.183 ± 2.597^a^	175.583 ± 4.539^d^	585.566 ± 2.396^c^	735.700 ± 2.848^b^
	LSD 0.05 = 10.754				
	*t*-test	—	117.11^*∗∗∗*^	−70.70^*∗∗∗*^	−90.93^*∗∗∗*^

Glutathione reductase (GSST) U/g kidney tissue	Mean ± SE	826.200 ± 2.755^a^	315.683 ± 3.560^d^	623.600 ± 2.735^c^	719.600 ± 2.848^b^
	LSD 0.05 = 7.978				
	*t*-test	—	109.14^*∗∗∗*^	−118.29^*∗∗∗*^	−119.70^*∗∗∗*^

Data are represented as mean ± SE. *∗∗∗*: significant at *P* < 0.001. For ANOVA analysis, within each row, means with different superscript (a, b, c, or d) are significantly different at *P* < 0.05, whereas means superscripts with the same letters mean that there is no significant difference at *P* < 0.05. LSD: least significant difference.

**Table 4:** Effect of administration of *L. sativum* and cinnamon methanol extracts for 4 weeks on lipid peroxide in the serum and kidney tissue homogenate in diabetic male rats.

Parameters	Statistics	G1 Negative control	G2 Positive control	G3 *L. sativum* extract	G4 Cinnamon extract
MDA nmol/mL	Mean ± SE	0.936 ± 0.035^d^	4.500 ± 0.057^a^	3.601 ± 0.061^b^	2.240 ± 0.038^c^
	LSD 0.05 = 0.164				
	*t*-test	—	−52.66^*∗∗∗*^	8.71^*∗∗∗*^	34.09^*∗∗∗*^

MDA nmol/g kidney tissue	Mean ± SE	2.586 ± 0.069^d^	16.081 ± 0.183^a^	7.581 ± 0.071^b^	4.731 ± 0.090^c^
	LSD 0.05 = 0.347				
	*t*-test	—	−70.12^*∗∗∗*^	36.50^*∗∗∗*^	51.68^*∗∗∗*^

Data are represented as mean ± SE. For *t*-test values, *∗∗∗* is significant at *P* < 0.001. For ANOVA analysis, within each row, means with different superscript (a, b, c, or d) are significantly different at *P* < 0.05, whereas means superscripts with the same letters mean that there is no significant difference at *P* < 0.05. LSD: least significant difference.

**Table 5:** Effect of administration of *L. sativum* and cinnamon methanol extracts for 4 weeks on serum urea, creatinine, uric acid, Na^+^, and P^+^ in diabetic rats.

Parameters mg/dL	Statistics	G1 Negative control	G2 Positive control	G3 *L. sativum* extract	G4 Cinnamon extract
Urea	Mean ± SE	24.50 ± 1.11^d^	74.83 ± 0.87^a^	46.66 ± 0.88^b^	36.83 ± 0.60^c^
	LSD 0.05 = 2.720				
	*t*-test	—	−29.16^*∗∗∗*^	24.75^*∗∗∗*^	28.32^*∗∗∗*^

Creatinine	Mean ± SE	0.68 ± 0.03^d^	3.63 ± 0.18^a^	2.78 ± 0.07^b^	1.62 ± 0.30^c^
	LSD 0.05 = 0.547				
	*t*-test	—	−14.90^*∗∗∗*^	3.47^*∗∗∗*^	7.12^*∗∗∗*^

Uric acid	Mean ± SE	3.33 ± 0.08^d^	6.68 ± 0.04^a^	5.60 ± 0.05^b^	4.70 ± 0.07^c^
	LSD 0.05 = 0.147				
	*t*-test	—	−59.53^*∗∗∗*^	13.67^*∗∗∗*^	23.80^*∗∗∗*^

Sodium	Mean ± SE	143.833 ± 0.945^a^	118.333 ± 0.881^d^	125.833 ± 0.477^c^	133.000 ± 1.154^b^
	LSD 0.05 = 3.523				
	*t*-test	—	19.85^*∗∗∗*^	−7.83^*∗∗∗*^	−8.06^*∗∗∗*^

Potassium	Mean ± SE	4.866 ± 0.033^a^	3.033 ± 0.088^d^	3.516 ± 0.054^c^	4.183 ± 0.060^b^
	LSD 0.05 = 0.392				
	*t*-test	—	17.39^*∗∗∗*^	−5.54^*∗∗∗*^	−10.88^*∗∗∗*^

Data are represented as mean ± SE. For *t*-test values, *∗∗∗* is significant at *P* < 0.001. For ANOVA analysis, within each row, means with different superscript (a, b, c, or d) are significantly different at *P* < 0.05, whereas means superscripts with the same letters mean that there is no significant difference at *P* < 0.05. LSD: least significant difference.

**Table 6:** Effect of administration of *L. sativum* and cinnamon methanol extracts for 4 weeks on urine albumin and creatinine in diabetic rats.

Parameters mg/dL	Statistics	G1 Negative control	G2 Positive control	G3 *L. sativum* extract	G4 Cinnamon extract
Urine albumin	Mean ± SE	22.16 ± 1.70^d^	411.50 ± 7.74^a^	291.50 ± 2.36^b^	163.50 ± 2.98^c^
	LSD 0.05 = 13.638				
	*t*-test	—	−47.24^*∗∗∗*^	16.17^*∗∗∗*^	24.64^*∗∗∗*^

Urine creatinine	Mean ± SE	85.00 ± 0.85^a^	27.00 ± 0.36^d^	45.83 ± 1.57^c^	62.16 ± 1.19^b^
	LSD 0.05 = 3.148				
	*t*-test	—	84.90^*∗∗∗*^	−11.61^*∗∗∗*^	−37.18^*∗∗∗*^

Data are represented as mean ± SE. For *t*-test values, *∗∗∗* is significant at *P* < 0.001. For ANOVA analysis, within each row, means with different superscript (a, b, c, or d) are significantly different at *P* < 0.05, whereas means superscripts with the same letters mean that there is no significant difference at *P* < 0.05. LSD: least significant difference.

**Table 7:** Effect of administration of *L. sativum* and cinnamon methanol extracts for 4 weeks on serum immunoglobulins in diabetic rats.

Immunoglobulins mg/dL	Statistics	G1 Negative control	G2 Positive control	G3 *L. sativum* extract	G4 Cinnamon extract
IgG	Mean ± SE	530.66 ± 1.05^d^	754.33 ± 3.46^a^	702.00 ± 3.04^b^	581.16 ± 1.66^c^
	LSD 0.05 = 6.952				
	*t*-test	—	−63.91^*∗∗∗*^	14.09^*∗∗∗*^	52.62^*∗∗∗*^

IgA	Mean ± SE	99.16 ± 1.88^d^	359.83 ± 1.74^a^	267.66 ± 2.74^b^	135.50 ± 1.28^c^
	LSD 0.05 = 6.261				
	*t*-test	—	−85.42^*∗∗∗*^	31.37^*∗∗∗*^	159.51^*∗∗∗*^

IgM	Mean ± SE	129.83 ± 1.07^d^	357.16 ± 2.24^b^	250.33 ± 1.60^c^	581.16 ± 1.66^a^
	LSD 0.05 = 4.776				
	*t*-test	—	−138.06^*∗∗∗*^	51.15^*∗∗∗*^	−81.25^*∗∗∗*^

Data are represented as mean ± SE. For *t*-test values, *∗∗∗* is significant at *P* < 0.001. For ANOVA analysis, within each row, means with different superscript (a, b, c, or d) are significantly different at *P* < 0.05, whereas means superscripts with the same letters mean that there is no significant difference at *P* < 0.05. LSD: least significant difference.

**Table 8:** Effect of administration of *L. sativum* and cinnamon methanol extracts for 4 weeks on the total body weight in diabetic rats.

Total body weight (g)	Statistics	G1 Negative control	G2 Positive control	G3 *L. sativum* extract	G4 Cinnamon extract
1st week	Mean ± SE	188.83 ± 0.40^c^	182.83 ± 0.79^d^	194.83 ± 0.83^b^	201.66 ± 0.66^a^
	LSD 0.05 = 1.922				
	*t*-test	—	8.78^*∗∗∗*^	−17.56^*∗∗∗*^	−19.21^*∗∗∗*^

2nd week	Mean ± SE	194.00 ± 0.81^c^	187.16 ± 0.79^d^	202.50 ± 0.76^b^	210.66 ± 0.42^a^
	LSD 0.05 = 2.276				
	*t*-test	—	4.72^*∗∗∗*^	−20.17^*∗∗∗*^	−41.76^*∗∗∗*^

3rd week	Mean ± SE	207.83 ± 0.70^b^	192.33 ± 1.08^c^	210.16 ± 0.54^b^	215.00 ± 0.77^a^
	LSD 0.05 = 2.349				
	*t*-test	—	10.82^*∗∗∗*^	−15.28^*∗∗∗*^	−28.23^*∗∗∗*^

4th week	Mean ± SE	210.33 ± 0.91^c^	194.00 ± 1.69^d^	213.66 ± 0.61^b^	219.00 ± 0.44^a^
	LSD 0.05 = 3.091				
	*t*-test	—	7.32^*∗∗∗*^	−10.30^*∗∗∗*^	−17.11^*∗∗∗*^

Data are represented as mean ± SE. For *t*-test values, *∗∗∗* is significant at *P* < 0.001. For ANOVA analysis, within each row, means with different superscript (a, b, c, or d) are significantly different at *P* < 0.05, whereas means superscripts with the same letters mean that there is no significant difference at *P* < 0.05. LSD: least significant difference.

**Table 9:** Effect of administration of *L. sativum* and cinnamon methanol extracts for 4 weeks on food intake in diabetic rats.

Food intake g/day	Statistics	G1 Negative control	G2 Positive control	G3 *L. sativum* extract	G4 Cinnamon extract
1st week	Mean ± SE	14.50 ± 0.22^a^	14.50 ± 0.22^a^	14.66 ± 0.21^a^	14.50 ± 0.22^a^
	LSD 0.05 = 0.698				
	*t*-test	—	0.00^NS^	−0.41^NS^	0.00^NS^

2nd week	Mean ± SE	16.66 ± 0.21^a^	16.66 ± 0.21^a^	16.50 ± 0.22^a^	16.50 ± 0.22^a^
	LSD 0.05 = 0.622				
	*t*-test	—	0.00^NS^	0.54^NS^	0.54^NS^

3rd week	Mean ± SE	16.66 ± 0.21^a^	16.50 ± 0.22^a^	16.83 ± 0.30^a^	16.66 ± 0.21^a^
	LSD 0.05 = 0.686				
	*t*-test	—	0.54^NS^	−1.00^NS^	−0.54^NS^

4th week	Mean ± SE	17.66 ± 0.21^a^	18.66 ± 0.42^a^	19.00 ± 0.44^a^	18.66 ± 0.42^a^
	LSD 0.05 = 1.329				
	*t*-test	—	−1.93^NS^	−0.41^NS^	0.00^NS^

Food intake g/day	Mean ± SE	16.37 ± 0.26^a^	16.58 ± 0.33^a^	16.75 ± 0.35^a^	16.58 ± 0.33^a^
	LSD 0.05 = 0.925	—	−1.04^NS^	−0.70^NS^	0.00^NS^
	*t*-test				

Data are represented as mean ± SE. For *t*-test values, *∗* is significant at *P* < 0.05, *∗∗* is significant at *P* < 0.01, and *∗∗∗* is significant at *P* < 0.001. For ANOVA analysis, within each row, means with different superscript (a, b, c, or d) are significantly different at *P* < 0.05, whereas means superscripts with the same letters mean that there is no significant difference at *P* < 0.05. LSD: least significant difference, NS: nonsignificant.

**Table 10:** Effect of administration of *L. sativum* and cinnamon methanol extracts for 4 weeks on water consumption by diabetic rats.

Water consumed mL/day	Statistics	G1 Negative control	G2 Positive control	G3 *L. sativum* extract	G4 Cinnamon extract
1st week	Mean ± SE	33.33 ± 1.05^ab^	32.50 ± 1.11^b^	36.33 ± 0.88^a^	36.33 ± 0.88^a^
	LSD 0.05 = 3.257				
	*t*-test	—	0.415^NS^	−4.60^*∗∗∗*^	−2.49^*∗∗*^

2nd week	Mean ± SE	33.8 ± 1.24^b^	37.16 ± 0.79^a^	36.83 ± 0.87^a^	36.16 ± 1.40^ab^
	LSD 0.05 = 2.578				
	*t*-test	—	−2.65^*∗∗*^	0.26^NS^	0.69^NS^

3rd week	Mean ± SE	34.66 ± 1.05^a^	36.83 ± 0.87^a^	37.50 ± 0.92^a^	37.16 ± 0.79^a^
	LSD 0.05 = 3.035				
	*t*-test	—	−2.13^*∗*^	−0.38^NS^	−0.21^NS^

4th week	Mean ± SE	29.16 ± 1.53^a^	29.66 ± 1.33^a^	33.33 ± 1.54^a^	33.00 ± 1.91^a^
	LSD 0.05 = 5.155				
	*t*-test	—	−0.24^NS^	−1.67^NS^	−1.28^NS^

Data are represented as mean ± SE. For *t*-test values *∗* is significant at *P* < 0.05, *∗∗* is significant at *P* < 0.01, and *∗∗∗* is significant at *P* < 0.001. For ANOVA analysis, within each row, means with different superscript (a, b, c, or d) are significantly different at *P* < 0.05, whereas means superscripts with the same letters mean that there is no significant difference at *P* < 0.05. LSD: least significant difference; NS: nonsignificant.

**Table 11:** Effect of administration of *L. sativum* and cinnamon methanol extracts for 4 weeks on organ weight in diabetic rats.

Organs weight, g	Statistics	G1 Negative control	G2 Positive control	G3 *L. sativum* extract	G4 Cinnamon extract
Heart	Mean ± SE	0.433 ± 0.091^a^	0.450 ± 0.018^a^	0.550 ± 0.022^a^	0.566 ± 0.021^a^
	LSD 0.05 = 0.127				
	*t*-test	—	−0.20^NS^	−3.46^*∗∗*^	−11.06^*∗∗∗*^

Liver	Mean ± SE	4.100 ± 0.821^a^	4.866 ± 0.084^a^	5.383 ± 0.153^a^	5.200 ± 0.077^a^
	LSD 0.05 = 1.290				
	*t*-test	—	−0.94^NS^	−2.79^*∗∗*^	−2.59^*∗∗*^

Right kidney	Mean ± SE	0.483 ± 0.101^b^	0.633 ± 0.021^ab^	0.666 ± 0.021^a^	0.633 ± 0.021^ab^
	LSD 0.05 = 0.151				
	*t*-test	—	−1.62^NS^	−1.00^NS^	0.00^NS^

Left kidney	Mean ± SE	0.516 ± 0.104^a^	0.683 ± 0.016^a^	0.633 ± 0.021^a^	0.633 ± 0.021^a^
	LSD 0.05 = 0.168				
	*t*-test	—	−1.53^NS^	2.23^*∗*^	1.46^NS^

Right testes	Mean ± SE	0.933 ± 0.194^a^	0.965 ± 0.020^a^	1.133 ± 0.021^a^	1.133 ± 0.021^a^
	LSD 0.05 = 0.299				
	*t*-test	—	−0.15^NS^	−4.99^*∗∗∗*^	−7.76^*∗∗∗*^

Left testes	Mean ± SE	0.966 ± 0.201^a^	0.956 ± 0.019^a^	1.1833 ± 0.030^a^	1.1833 ± 0.016^a^
	LSD 0.05 = 0.298				
	*t*-test	—	0.04^NS^	−5.14^*∗∗∗*^	−7.37^*∗∗∗*^

Data are represented as mean ± SE. For *t*-test values, *∗* is significant at *P* < 0.05, *∗∗* is significant at *P* < 0.01, and *∗∗∗* is significant at *P* < 0.001. For ANOVA analysis, within each row, means with different superscript (a, b, c, or d) are significantly different at *P* < 0.05, whereas means superscripts with the same letters mean that there is no significant difference at *P* < 0.05. LSD: least significant difference; NS: nonsignificant.

**Table 12:** Effect of administration of *L. sativum* and cinnamon methanol extracts for 4 weeks on food intake (FI), body weight gain (BWG), and food efficiency ratio (FER) in diabetic rats.

Biological evaluation	Statistics	G1 Negative control	G2 Positive control	G3 *L. sativum* extract	G4 Cinnamon extract
BWG g/day	Mean ± SE	0.822 ± 0.026^a^	0.422 ± 0.061^b^	0.755 ± 0.014^a^	0.738 ± 0.035^a^
	LSD 0.05 = 3.379				
	*t*-test	—	5.13^*∗∗∗*^	−5.77^*∗∗∗*^	−9.58^*∗∗∗*^

BWG g/4 weeks	Mean ± SE	24.666 ± 0.802^a^	12.666 ± 1.855^b^	22.666 ± 0.421^a^	22.166 ± 1.077^a^
	LSD 0.05 = 0.153				
	*t*-test	—	5.13^*∗∗∗*^	−5.77^*∗∗∗*^	−9.58^*∗∗∗*^

BWG %	Mean ± SE	13.284 ± 0.425^a^	6.991 ± 1.029^c^	11.867 ± 0.223^ab^	11.274 ± 0.592^b^
	LSD 0.05 = 1.792				
	*t*-test	—	4.91^*∗∗∗*^	−5.18^*∗∗∗*^	−7.76^*∗∗∗*^

FER g/day	Mean ± SE	0.050 ± 0.001^a^	0.025 ± 0.003^b^	0.045 ± 0.000^a^	0.044 ± 0.002^a^
	LSD 0.05 = 0.006				
	*t*-test	—	5.34^*∗∗∗*^	−5.77^*∗∗∗*^	−9.58^*∗∗∗*^

FER%	Mean ± SE	5.022 ± 0.163^a^	2.546 ± 0.373^b^	4.510 ± 0.083^a^	4.456 ± 0.216^a^
	LSD 0.05 = 0.127				
	*t*-test	—	5.24^*∗∗∗*^	−5.63^*∗∗∗*^	−9.57^*∗∗∗*^

Data are represented as mean ± SE. For *t*-test values, *∗* is significant at *P* < 0.05, *∗∗* is significant at *P* < 0.01, and *∗∗∗* is significant at *P* < 0.001. For ANOVA analysis, within each row, means with different superscript (a, b, c, or d) are significantly different at *P* < 0.05, whereas means superscripts with the same letters mean that there is no significant difference at *P* < 0.05. LSD: least significant difference; NS: nonsignificant.

## Abbreviations

AGEs: Advanced glycation end products
ATP: Adenosine triphosphate
BWG: Body weight gain
CAT: Catalase
CML: Carboxymethyl lysine
CRE: Creatinine
DM: Diabetes mellitus
FBS: Fasting blood sugar
FER: Food efficiency ratio
FI: Food intake
g/dL: Gram per deciliter
G1: Negative control group
G2: Hyperglycemic positive control group
G3: Hyperglycemic rats treated with 20% cress seeds methanol extract
G4: Hyperglycemic rats treated with 20% cinnamon methanol extract
GST: Glutathione S-transferase
IgA: Immunoglobulin A
IgG: Immunoglobulin G
IgM: Immunoglobulin M
IGT: Impaired glucose tolerance
IL-6: Interleukin-6
LS: Lepidium sativum
MDA: Malondialdehyde
mg/dL: Milligram per deciliter
mmol/L: Millimole per liter
MODY: Maturity-onset diabetes of the young.
ROS: Reactive oxygen species
SOD: Superoxide dismutase
STZ: Streptozotocin
U/g: Unit per gram
U/L: Unit per liter
U/mL: Unit per milliliter
UA: Uric acid
w/w: Weight to weight ratio
*μ*mol/L: Micromole per liter.

## References

[1] Ping H., Zhang G., Ren G. (2010). Antidiabetic effects of cinnamon oil in diabetic KK-Ay mice. *Food and Chemical Toxicology*.

[2] Ranasinghe P., Jayawardana R., Galappaththy P., Constantine G. R., de Vas Gunawardana N., Katulanda P. (2012). Efficacy and safety of ‘true’ cinnamon (*Cinnamomum zeylanicum*) as a pharmaceutical agent in diabetes: a systematic review and meta-analysis. *Diabetic Medicine*.

[3] Cazzola R., Estaro B. (2014). Antioxidant spices and herbs used in diabetes. *Diabetes*.

[4] Prabhakar P. K., Doble M. (2011). Mechanism of action of natural products used in the treatment of diabetes mellitus. *Chinese Journal of Integrative Medicine*.

[5] Lu T., Sheng H., Wu J., Cheng Y., Zhu J., Chen Y. (2012). Cinnamon extract improves fasting blood glucose and glycosylated hemoglobin level in Chinese patients with type 2 diabetes. *Nutrition Research*.

[6] Ranilla L. G., Kwon Y.-I., Apostolidis E., Shetty K. (2010). Phenolic compounds, antioxidant activity and in vitro inhibitory potential against key enzymes relevant for hyperglycemia and hypertension of commonly used medicinal plants, herbs and spices in Latin America. *Bioresource Technology*.

[7] Eddouks M., Maghrani M., Zeggwagh N.-A., Michel J. B. (2005). Study of the hypoglycaemic activity of *Lepidium sativum* L. aqueous extract in normal and diabetic rats. *Journal of Ethnopharmacology*.

[8] Abdelwahab S. I., Mariod A. A., Taha M. M. E. (2014). Chemical composition and antioxidant properties of the essential oil of *Cinnamomum altissimum* Kosterm. (Lauraceae). *Arabian Journal of Chemistry*.

[9] Khataibeh M. (2013). Cinnamon modulates biochemical alterations in rats loaded with acute restraint stress. *Journal of Saudi Chemical Society*.

[10] Sartorius T., Peter A., Schulz N. (2014). Cinnamon extract improves insulin sensitivity in the brain and lowers liver fat in mouse models of obesity. *PLoS ONE*.

[11] Prajapati V. D., Maheriya P. M., Jani G. K., Patil P. D., Patel B. N. (2014). *Lepidium sativum* Linn.: a current addition to the family of mucilage and its applications. *International Journal of Biological Macromolecules*.

[12] Behrouzian F., Razavi S. M. A., Phillips G. O. (2014). Cress seed (*Lepidium sativum*) mucilage, an overview. *Bioactive Carbohydrates and Dietary Fibre*.

[13] Hassan L. K., Haggag H., ElKalyoubi M., Abd EL-Aziz M., El-Sayed M., Sayed A. (2015). Physico-chemical properties of yoghurt containing cress seed mucilage or guar gum. *Annals of Agricultural Sciences*.

[14] Bansal D., Bhasin P., Yadav O., Punia A. (2012). Assessment of genetic diversity in *Lepidium sativum* (Chandrasur) a medicinal herb used in folklore remedies in India using RAPD. *Journal of Genetic Engineering and Biotechnology*.

[15] Sayed A. A. R. (2012). Ferulsinaic acid modulates SOD, GSH, and antioxidant enzymes in diabetic kidney. *Evidence-Based Complementary and Alternative Medicine*.

[16] Malick C. P., Singh M. B. (1980). *Plant Enzymology and Histo Enzymology Kalyari*.

[17] Dubois M., Gilles K. A., Hamilton J. K., Rebers P. A., Smith F. (1956). Colorimetric method for determination of sugars and related substances. *Analytical Chemistry*.

[18] Adebayo A., Ishola R., Taiwo S., Majolagbe N., Adekeye T. (2009). Evaluations of the methanol extract of *Ficus exasperata* stem bark, leaf and root for phytochemical analysis and antimicrobial. *African Journal of Plant Science*.

[19] Al-Malki A. L., El Rabey H. A. (2015). The antidiabetic effect of low doses of *Moringa oleifera* lam. Seeds on streptozotocin induced diabetes and diabetic nephropathy in male rats. *BioMed Research International*.

[20] Barham D., Trinder P. (1972). GOD-PAP enzymatic colorimetric method of glucose estimation without deproteinization. *Analyst*.

[21] Monnier V. M., Cerami A. (1981). Nonenzymatic browning in vivo: possible process for aging of long-lived proteins. *Science*.

[22] Nishikimi M., Rao N. A., Yagi K. (1972). The occurrence of superoxide anion in the reaction of reduced phenazine methosulfate and molecular oxygen. *Biochemical and Biophysical Research Communications*.

[23] Aebi H. (1984). Catalase in vitro. *Methods in Enzymology*.

[24] Habig W. H., Pabst M. J., Jakoby W. B. (1974). Glutathione S transferases. The first enzymatic step in mercapturic acid formation. *Journal of Biological Chemistry*.

[25] Ohkawa H., Ohishi N., Yagi K. (1979). Assay for lipid peroxides in animal tissues by thiobarbituric acid reaction. *Analytical Biochemistry*.

[26] Bartels H., Böhmer M., Heierli C. (1972). Serum creatinine determination without protein precipitation. *Clinica Chimica Acta*.

[27] Berthelot M. (1859). Berthelot's reaction mechanism. *Report de Chimie Applique*.

[28] Fawcett J. K., Scott J. E. (1960). A rapid and precise method for the determination of urea. *Journal of Clinical Pathology*.

[29] Trinder P. (1951). A rapid method for the determination of sodium in serum. *The Analyst*.

[30] Terri A., Sesin P. (1958). Determination of serum potassium by using sodium tetraphenylboro method. *American Journal of Clinical Pathology*.

[31] Berne G. (1974). Detection of total IgG. *Clinical Chemistry*.

[32] Davies B., Morris T. (1993). Physiological parameters in laboratory animals and humans. *Pharmaceutical Research*.

[33] Drury R., Wallington E., Cancerson R. (1976). *Carleton's Histological Technique*.

[34] Qrtmeyer H. K. (1997). Mechanism of in vivo insulin action on liver glycogen synthase includes activation of protein phosphatase 2C in Rhesus monkeys. *Experimental and Clinical Endocrinology and Diabetes*.

[35] Scalbert A., Manach C., Morand C., Rémésy C., Jiménez L. (2005). Dietary polyphenols and the prevention of diseases. *Critical Reviews in Food Science and Nutrition*.

[36] Akilen R., Tsiami A., Robinson N. (2013). Efficacy and safety of ‘true’ cinnamon (*Cinnamomum zeylanicum*) as a pharmaceutical agent in diabetes: a systematic review and meta-analysis. *Diabetic Medicine*.

[37] Hamidpour R., Hamidpour M., Hamidpour S., Shahlari M. (2015). Cinnamon from the selection of traditional applications to its novel effects on the inhibition of angiogenesis in cancer cells and prevention of Alzheimer's disease, and a series of functions such as antioxidant, anticholesterol, antidiabetes, antibacterial, antifungal, nematicidal, acaracidal, and repellent activities. *Journal of Traditional and Complementary Medicine*.

[38] Wellen K. E., Hotamisligil G. S. (2005). Inflammation, stress, and diabetes. *Journal of Clinical Investigation*.

[39] Li R. W., Theriault A. G., Au K. (2006). Citrus polymethoxylated flavones improve lipid and glucose homeostasis and modulate adipocytokines in fructose-induced insulin resistant hamsters. *Life Sciences*.

[40] Adisakwattana S., Sompong W., Meeprom A., Ngamukote S., Yibchok-Anun S. (2012). Cinnamic acid and its derivatives inhibit fructose-mediated protein glycation. *International Journal of Molecular Sciences*.

[41] Eidi A., Mortazavi P., Bazargan M., Zaringhalam J. (2012). Hepatoprotective activity of cinnamon ethanolic extract against CCL 4-induced liver injury in rats. *Experimental and Clinical Sciences Journal*.

[42] Baynes J., Thorpe S. (1999). Role of oxidative stress in diabetic complications: a new perspective on an old paradigm. *Diabetes*.

[43] Dugoua J.-J., Seely D., Perri D. (2007). From type 2 diabetes to antioxidant activity: a systematic review of the safety and efficacy of common and cassia cinnamon bark. *Canadian Journal of Physiology and Pharmacology*.

[44] Ghosh T., Basu A., Adhikari D., Roy D., Pal A. K. (2015). Antioxidant activity and structural features of *Cinnamomum zeylanicum*. *3 Biotech*.

[45] Mogensen C. E., Christensen C. K. (1985). Blood pressure changes and renal function in incipient and overt diabetic nephropathy. *Hypertension*.

[46] Kumar K., Issac A., Ninan E., Kuttan R., Maliakel B. (2014). Enhanced anti-diabetic activity of polyphenol-rich de-coumarinated extracts of *Cinnamomum cassia*. *Journal of Functional Foods*.

[47] Guang-Wei L., Katsuyuki M., Tokihito Y., Kenjiro Y. (1994). Effects of extract from *Clerodendron trichotomum* on blood pressure and renal function in rats and dogs. *Journal of Ethnopharmacology*.

[48] Kreydiyyeh S. I., Usta J., Copti R. (2000). Effect of cinnamon, clove and some of their constituents on the Na^+^-K^+^-ATPase activity and alanine absorption in the rat jejunum. *Food and Chemical Toxicology*.

[49] Diwakar B., Dutta P., Lokesh B., Naidu K. (2010). Physicochemical properties of garden cress (*lepidium sativum* l.) seed oil. *Journal of the American Oil Chemists' Society*.

[50] Muthenna P., Raghu G., Kumar P. A., Surekha M. V., Reddy G. B. (2014). Effect of cinnamon and its procyanidin-B2 enriched fraction on diabetic nephropathy in rats. *Chemico-Biological Interactions*.

[51] Tung Y.-T., Chua M.-T., Wang S.-Y., Chang S.-T. (2008). Anti-inflammation activities of essential oil and its constituents from indigenous cinnamon (*Cinnamomum osmophloeum*) twigs. *Bioresource Technology*.

[52] White A., Nunes C., Escudier M. (2006). Improvement in orofacial granulomatosis on a cinnamon- and benzoate-free diet. *Inflammatory Bowel Diseases*.

[53] Beejmohun V., Peytavy-Izard M., Mignon C. (2014). Acute effect of Ceylon cinnamon extract on postprandial glycemia: alpha-amylase inhibition, starch tolerance test in rats, and randomized crossover clinical trial in healthy volunteers. *BMC Complementary and Alternative Medicine*.

[54] Elgawish R. A. R., Abdelrazek H. M. A. (2014). Effects of lead acetate on testicular function and caspase-3 expression with respect to the protective effect of cinnamon in albino rats. *Toxicology Reports*.

[55] Torki M., Akbari M., Kaviani K. (2014). Single and combined effects of zinc and cinnamon essential oil in diet on productive performance, egg quality traits, and blood parameters of laying hens reared under cold stress condition. *International Journal of Biometeorology*.

[56] Al-Yahya M., Mossa J., Ageel A., Rafatullah S. (1994). Pharmacological and safety evaluation studies on *Lepidium sativum* L., Seeds. *Phytomedicine*.

[57] Ullah N., Khan M. A., Khan T., Ahmad W. (2012). Bioactive traditional plant *Cinnamomum zeylanicum* successfully combat against nephrotoxic effects of aminoglycosides. *Bangladesh Journal of Pharmacology*.

